# Unchanging dynamics in posttraumatic growth in cancer patients: ways of coping and illness perception

**DOI:** 10.3389/fpsyg.2023.1223131

**Published:** 2023-09-01

**Authors:** Seda Bayraktar, Mine Ozkan

**Affiliations:** ^1^Faculty of Literature, Department of Psychology, Akdeniz University, Antalya, Türkiye,; ^2^Faculty of Medicine, Department of Psychiatry, Istanbul University, Istanbul, Türkiye

**Keywords:** cancer, psychological trauma, posttraumatic growth, ways of coping, illness perception

## Abstract

**Introduction:**

This study aims to address the positive changes due to traumatic experiences, such as being diagnosed with cancer and experiencing this disease for a certain period. Within this purpose, socio-demographic and disease-related variables, coping ways and illness perceptions that affect posttraumatic growth in cancer patients were examined. Secondly, the findings of this study, which is one of the first studies on posttraumatic growth in cancer patients in Turkey, were compared with the findings of current studies on the subject.

**Method:**

Datums were collected by an interview form and three scales (Posttraumatic Growth Scale, Ways of Coping Inventory and Illness Perception Scale-R) to 78 cancer outpatients in Istanbul University Oncology Institute in 2007.

**Results:**

Results showed that cancer patients have higher posttraumatic growth levels than the mean. According to analysis, posttraumatic growth total score between confrontive coping (*t* = −2.344, *p* < 0.05), self-controlling (*t* = −3.704, *p* < 0.001), accepting responsibility (*t* = −3.032, *p* < 0.01), escape-avoidance (*t* = −2.285, *p* < 0.05), planful problem solving (*t* = −2.502, *p* < 0.05), positive reappraisal (*t* = −5.241, *p* < 0.001), and seeking social support (*t* = −3.527, *p* < 0.01) has relationship. Also, there is a relation between posttraumatic growth subscales and the Revised form of Illness Perception Questionnaire; Change in relationships with others subscale (*t* = 2.887, *p* < 0.01) and Change in self-perception subscale (*t* = 2.660, *p* < 0.01) between timeline (acute/chronic), Change in self-perception subscale between timeline (cyclical) (*t* = −2.788, *p* < 0.01) and uncontrollable body factors (*t* = −1.916, *p* < 0.05) Change in philosophy of life subscale between external attributions (*t* = −2.057, *p* < 0.05) and Change in relationships with others subscale (*t* = −2.920, *p* < 0.01) between chance factors. It was found that positive reappraisal (*F* = 78.290, *p* < 0.001), self-controlling (*F* = 39.814, *p* < 0.001), and distancing (*F* = 46.311, *p* < 0.001) were significant predictors of posttraumatic growth total score. Results showed that ways of coping and illness perceptions were essential variables in posttraumatic growth.

**Discussion:**

Studies on posttraumatic growth in Turkey and the world have significantly increased in recent years. This study aimed to examine the findings obtained from cancer patients in 2007 in discussion with the findings in the current literature. In this context, it is seen that the relevant variables affecting posttraumatic growth in cancer patients in different cultures do not change.

## 1. Introduction

Traumatic events can generally be classified as incidents caused by human actions and events concerning other factors. Events caused by human actions include sexual assault, physical violence, and similar occurrences, while events not caused by human actions are classified as natural disasters, diseases, and accidents. It is known in the relevant literature that events caused by human actions have a higher likelihood of causing various psychiatric problems, particularly post-traumatic stress disorder (PTSD). Among traumatic experiences that humans do not intentionally cause, cancer is important in psychological trauma studies (Türksoy, 2003; Dobrikova et al., 2021; Baník et al., 2022).

Cancer is one of humanities leading problems, especially in contemporary medicine, and evokes adverse reactions and thoughts, such as fear, hopelessness, helplessness, guilt, abandonment, and death. Cancer is a disease that should be evaluated holistically with medical, psychological, social, economic, and spiritual components (Özkan, 1993). Exposure to cancer is considered to be a traumatic experience due to its unusual, chronic, and unexpected nature (Tedeschi and Calhoun, 1995). Examination of the literature reveals that an increasing amount of information claims that positive changes can arise out of the negative consequences of different types of traumas, especially in cancer patients; the growth phenomenon perceived after traumatic experiences involving high levels of stress is called posttraumatic growth (PTG) (Tedeschi et al., 2018; Faustova, 2020; Baník et al., 2022; Li, 2022). This study aimed to address the positive changes that occur as a result of being diagnosed with cancer and experiencing the disease for a certain period.

Posttraumatic growth is a term used to describe the positive changes experienced by individuals due to a struggle with life crises involving high levels of stress (Tedeschi and Calhoun, 2004; Tedeschi et al., 2018). Posttraumatic growth includes changes in three main areas: self-perceptions, relationships with others, and philosophy of life. According to the functional-descriptive model proposed by Calhoun and Tedeschi (1998), many variables, such as ways of coping, social support, ruminative thoughts, and personality traits, are important in the emergence of PTG (Calhoun et al., 2010; Calhoun and Tedeschi, 2013; Tedeschi et al., 2018). It can be seen from the literature that PTG, and its relationship with various variables, has been investigated in different types of traumas. For example, in fire-fighters (Armstrong et al., 2014; Sattler et al., 2014; Kehl et al., 2015), Hurricane Katrina survivors (Kilmer and Gil-Rivas, 2010; Chan and Rhodes, 2013), spinal cord injury patients (Chun and Lee, 2010; January et al., 2015), earthquake survivors (Karanci and Acartürk, 2005; Eren-Koçak and Kılıç, 2014; Taku et al., 2015), and accident survivors (Rabe et al., 2006; Zoellner et al., 2008).

Most of the research on PTG in health psychology has been conducted with cancer patients (Cordova et al., 2001; Lechner et al., 2003; Sears et al., 2003; Ho et al., 2004; Widows et al., 2005; Thornton and Perez, 2006; Liu et al., 2020; Aydoğdu and Dirik, 2021) and it has been shown that positive health behaviors in many patients are due to significant cognitive restructuring (Tedeschi et al., 2018). In this context, it is considered important to examine two concepts: ways of coping and illness perceptions. However, in the relevant literature, no studies have examined ways of coping and illness perceptions together in the context of PTG in cancer patients; this study addresses this gap, therefore.

In general, in the current literature, studies of PTG in cancer patients have shown that ways of coping, religion, and traumatic stress are predictors of PTG in women diagnosed with ovarian cancer (Oh et al., 2021), cancer patients with high religious and spiritual beliefs show higher levels of PTG (Schwarz and Vavrová, 2021). Moreover, while social support is positively correlated with PTG in cancer patients who are receiving treatment or in remission, it is not correlated with PTG in those who are in the terminal stage of cancer and there is a positive correlation between self-efficacy and PTG in treatment (remission) and terminal stage cancer patients (Dobrikova et al., 2021). In a study examining locus of control, ways of coping, emotion regulation strategies, and social support in patients diagnosed with lung cancer, social support had a direct positive effect on PTG; ways of coping and cognitive reappraisal emotion regulation strategies were the main mediating variables and explained approximately 73.1% of the indirect effect between PTG and social support (Zhang et al., 2021).

Baghjari et al. (2017) reported that among problem-focused coping strategies, cognitive appraisal and seeking social support explained 53% of changes in PTG in women and men with advanced cancer and may be helpful in clinical interventions, such as problem-focused coping skills training and facilitative measures to provide social support. In their qualitative study, Lelorain et al. (2012) revealed that the PTG is a theme specific to women with high levels of coping, social support, and active cognitive processing skills.

In their meta-analysis, Wan et al. (2022a) examined the relationship between PTG and resilience in breast cancer patients (17 studies, including 4,156 breast cancer patients) and found a high positive correlation between PTG and resilience, while Adamkovic et al. (2022) examined the relationship between life satisfaction, PTG, ways of coping and resilience in cancer patients and found that increasing life satisfaction was strongly associated with resilience, moderately associated with ways of coping, and weakly associated with PTG. In a study conducted by Schmidt et al. (2012) involving 54 cancer patients, it was discovered that having a secure attachment style was closely linked to positive reframing, active coping, and religion. Furthermore, all three variables demonstrated associations with PTG. Regression analysis revealed that positive reframing and religion served as coping mechanisms that could mediate the relationship between having a secure attachment style and experiencing PTG.

In summary, ways of coping, social support, resilience, and life satisfaction are among the variables that have been studied in conjunction with PTG in various types and stages of cancer patients. In these studies, ways of coping and similar concepts such as emotion regulation are frequently addressed when exploring PTG in cancer patients. However, illness perceptions have been less studied. Studies of illness perceptions frequently with ways of coping in cancer patients include:

Postolica et al. (2017) examined ways of coping, illness perceptions, and family adaptation to the disease in cancer patients with and without a family history of cancer, Kocyigit et al. (2021) examined illness perceptions, ways of coping, and magical thinking in breast cancer patients, Hopman and Rijken (2015) examined illness perceptions, the characteristics of illness, and ways of coping in cancer patients. In a similar way Kugbey et al. (2020) in women diagnosed with breast cancer in Ghana, Zhang et al. (2018) in patients diagnosed with lung cancer and Dempster et al. (2012) examined coping and illness perception in oesophageal cancer patients, while Krok et al. (2019) examined the mediating effect of meaning in life and ways of coping in the relationship between illness perceptions and affective symptoms in gastrointestinal cancer patients. However, it is seen that the concept of illness perception in cancer is also included in intervention methods. Stephenson et al. (2021) examined the role of coping and illness perceptions in supportive care in patients with various cancer diagnoses such as breast, colorectal, lung, prostate, and melanoma; Fischer et al. (2013) examined illness perceptions and coping in a psycho-educational group intervention for women with breast cancer. Pourfallahi et al. (2020) examined the effect of informational-emotional support programs on illness perception and emotional coping in patients diagnosed with breast, colorectal, gastrointestinal, lung and leukemia and undergoing chemotherapy.

Although studies that have addressed PTG and illness perceptions together are limited, some relevant findings have been reported. For example, Leal et al. (2016) examined PTG, core beliefs, and illness perceptions in women diagnosed with breast cancer using a structural equation model, while Banik (2012) examined PTG, psychological distress, and illness perceptions in people diagnosed with cancer. Rahimzadegan et al. (2022) examined the relationships between PTG, illness perceptions, and emotion regulation in cancer patients; they showed that negative illness perceptions were significantly and negatively related to PTG, while optimistic illness perceptions and emotion regulation skills were both significantly and positively related to PTG.

Lau et al. (2018) examined the relationship between illness perceptions and PTG in newly diagnosed HIV-positive men. Linear regression analyses conducted on the emotional representation subscale (*β* = −0.49) and five cognitive representation subscales (timeline, consequence, identity, attribution to God’s punishment/will, and attribution to chance/luck) revealed they were negatively correlated with PTG (*β* = −0.13 to −0.37), whereas four other cognitive representation subscales (coherence, treatment control, personal control, and attribution to carelessness) were positively correlated with PTG (*β* = 0.15 to 0.51). The associations between the five cognitive representation subscales and PTG were all mediated by emotional representation. The results suggest that interventions that promote PTG, especially ones that address illness perceptions and emotional representation, are necessary for this group of patients.

Rogan et al. (2013) conducted a study to examine how illness perceptions, distress, disability, ways of coping, and posttraumatic growth (PTG) were related in individuals with acquired brain injury (ABI). The findings revealed that individuals who reported higher levels of PTG were more likely to utilize adaptive coping strategies (*r* = 0.597), experience lower levels of distress (*r* = −0.241), and hold stronger beliefs about their ability to control the consequences of their brain injury through treatment (*r* = 0.263). Adaptive coping strategies were the most significant predictor of PTG (*sr2* = 0.287), explaining a large portion of the observed variance. Illness perceptions were not found to be significantly related to growth experiences. Our study is similar to Rogan et al.’s study in terms of evaluating PTG, illness perceptions, and ways of coping, but it uses a different patient group (cancer patients), and it is expected that different results will be found due to the different dynamics of the physical illnesses.

As a result of the increasing number of studies addressing PTG in cancer patient samples, and the increase in knowledge on the subject, systematic reviews and meta-analyses in this field have increased in recent years (e.g., Long et al., 2021; Ahmadi et al., 2022; Almeida et al., 2022; Knauer et al., 2022; Wan et al., 2022b; Wang et al., 2023). These studies, together with those of Lau et al. (2018) and Rogan et al. (2013), discussed above, indicate that cognitive and emotional factors are essential in facilitating PTG in cancer patients.

This study aimed to examine the phenomenon of PTG in cancer patients, and how it is related to ways of coping and illness perceptions, which are both thought to affect PTG. According to the number of studies and meta-analyses on PTG in cancer patients, it is seen that it is important to fill the gap in literature by examining ways of coping and individuals’ illness perceptions of their traumatic experiences, which are believed to be influential in positive growth following traumatic experiences, is particularly important for developing intervention approaches.

## 2. Materials and methods

### 2.1. Participants, procedure and aims of the study

This study was conducted with 78 cancer patients who applied to Istanbul University Oncology Institute as outpatients in 2007. The selection criteria for the sample group were that the participant should be literate, open to cooperation, willing to be interviewed, be aged from 18 to 65, have mental competence, did not have a psychotic disorder, and that at least 6 months and not more than 5 years had passed since the cancer diagnosis. This research aligns with the ethical principles outlined in the Declaration of Helsinki. It adheres to the ethical standards of the Istanbul University Faculty of Medicine, as confirmed by the institution’s ethics board approval number 2006/2123. The participants were recruited through convenience sampling, informed about the nature and purpose of the study, and signed the Voluntary Consent Form before participation.

In general, firstly, the level of PTG in cancer patients was determined and the effect of these variables on PTG was then examined. The frequencies and percentages for the relevant variables were examined in a Turkish sample of cancer patients who also self-reported the perceived causes of their disease via their responses on the Illness Perception Questionnaire. The research questions to be answered are as follows:

1. Do total and subscale PTG scores differ relative to the sociodemographic characteristics of cancer patients?
2. What is the frequency of occurrence of the total and subscale PTG scores of cancer patients?
3. Do the total and subscale PTG scores differ relative to the disesase related variables?
4. Is there a correlation between PTG scores, ways of coping scores and illness perception scores in cancer patients?
5. Is there a relationship between the ways of coping scores, total and subscale illness perception scores, and the High/Low total and subscale PTG scores of cancer patients?
6. Do the combined ways of coping and illness perception subscale scores predict the total and subscale PTG scores of cancer patients?
7. What are the subjective evaluations of the causes of cancer among cancer patients in Turkey?

### 2.2. Measures

A semi-structured interview form was used to collect socio-demographic and disease-related data. Three individual questionnaires were used to collect the data on PTG, ways of coping, and illness perceptions, which acted as the dependent and independent variables. These are discussed in turn below.

#### 2.2.1. Semi-structured interview form

This was self-authored and collected socio-demographic data (age, gender, marital status, etc.) and disease-related data (diagnosis, stage, treatment, etc.).

#### 2.2.2. Posttraumatic growth scale

The Posttraumatic Growth Scale (PTGS) was developed by Tedeschi and Calhoun (1996) to measure positive change due to traumatic events. Although there were 34 items in the first version of the scale, as a result of their analyses, the authors later transformed it into 21 items and five subscales: new possibilities, relating to others, personal strength, spiritual-existential change, and appreciation of life. The items are measured on a 6-point Likert-type scale (0 = *I have not experienced this change due to my life crisis* to 5 = *I have experienced this change a lot due to my life crisis*). An acceptable level of construct validity was found in university students, with an internal consistency coefficient of 0.90, and a test–retest reliability after two months of 0.71 (Cohen et al., 1998; Park and Lechner, 2006). Dirik (2006) made Turkish adaptation of the PTGS with rheumatoid arthritis patients and obtained three factors explaining 59% of the variance. These were named “Change in Relationships with Others,” “Change in Philosophy of Life,” and “Change in Self-Perception.” The overall reliability coefficient of the scale was 0.94. The scale used a 6-point Likert scale, as per the original scale. The Turkish adaptation by Dirik (2006) was used in the current study. The Cronbach’s alpha values of the PTGS obtained from the sample group in this study is 0.94 for total score, “Change in Relationships with Others 0.90, “Change in Philosophy of Life 0.81, and, 0.89 for the“Change in Self-Perception.”

#### 2.2.3. Ways of coping inventory

The Ways of Coping Inventory (WCI) was developed by Folkman and Lazarus (1985) to measure way of coping and consists of 66 items and eight subscales referring to a range of coping methods: confrontive coping (It refers to aggressive efforts to change the situation and involves hostility and willingness to take risks), distancing (It represents cognitive efforts to diminish the importance of the event and prevent personal impact), self-controlling (It includes individuals’ efforts to regulate their emotions and actions and bring them in order), seeking social support (It describes the efforts to seek informational, material, and emotional support), accepting responsibility (It refers to recognizing one’s role in resolving the problem and taking action to put things in order), escape-avoidance (It involves behavioral efforts to distance oneself from the problem and engage in wishful thinking), planful problem-solving (It describes problem-focused efforts used to change the situation, including an analytical approach to problem-solving), and positive reappraisal (It refers to efforts to find positive meaning in the situation based on personal development and may also involve a religious dimension). The WCI uses a 4-point Likert-type scale (0 not using-3 extensively using), which indicates how often each method is used (as cited in Kaçmaz, 2003). High Cronbach’s alphas have been reported for the Turkish adaptation of the WCI, which was used in this study (Kutlu, 1999: *α* = 0.83; Özkan and Kutlu, 2004, in their study with the relatives of patients with hematologic cancer: *α* = 0.92). The Cronbach’s alpha value of the WCI total score obtained from the sample group in this study is 0.93. The reliability coefficients of the subscales are as follows, in respectively: confrontive coping 0.54, distancing 0.66, self-controlling 0.56, seeking social support 0.79, accepting responsibility 0.58, escape-avoidance. 51, planful problem-solving 0.69, and positive reappraisal 0.76.

#### 2.2.4. Illness perception questionnaire-revised

The Illness Perception Questionnaire (IPQ) was developed by Weinman et al. (1996) and revised by Moss-Morris et al. (2002). The revised questionnaire consists of three dimensions: symptoms (identity), perceptions, and reasons. The *symptoms/identity* dimension is scored as “Yes = 1” and “No = 0” and consists of two subscales called identity A and identity B, each with 14 symptoms (e.g., pain, nausea, difficulty breathing, weight loss, fatigue, wheezing, headache, dizziness, difficulty sleeping, loss of strength, etc.). For each of these symptoms, the individual is first asked whether they have experienced it since the onset of the disease and then whether they associate this symptom with their illness. This dimension is designed in a format where the person responds yes/no to both questions. The sum of affirmative answers to the second question forms the evaluation result of the disease type dimension. The *perceptions* dimension is scored on a 5-point Likert-type scale and has 38 questions and seven subscales: timeline (acute/chronic), timeline (cyclical), consequences, personal control, treatment control, illness coherence, and emotional representations. Timeline subscales investigate the individual’s perceptions regarding the duration of their illness and classify it as acute, chronic, or cyclical. The consequences subscale explores the individual’s beliefs about the possible effects of their illness on severity, and physical, social, and psychological functionality. Personal control examines the individual’s internal perception of control over their illness’s duration, course, and treatment. Treatment control investigates the individual’s beliefs about the effectiveness of the applied treatment. Understanding of the disease assesses the individual’s understanding or grasp of their illness. Emotional representations explore the individual’s feelings related to their illness. The *reasons* dimension is also scored on a 5-point Likert-type scale and consists of 18 questions and five subscales: personal attributions (stress or anxiety, my attitude, personality traits, emotional state, family problems, decreased body resistance, my own behavior), external attributions (poor medical care in my past, environmental pollution, accident or injury, overwork), lifestyle attributions (smoking, alcohol, diet, eating habits), uncontrollable bodily attributions (a germ or virus, hereditary-irritable, aging), and chance attributions (luck or bad luck). In addition, there is a dimension requiring qualitative assessment whereby the individual is asked to write down the three most important reasons for his/her illness (Armay, 2006; Kocaman et al., 2007).

Armay (2006) examined the validity and reliability of the IPQ-R using cancer patients, finding Cronbach’s alphas for the various subscales from 0.604 to 0.859. With internal medicine clinic patients, Kocaman et al. (2007) found alpha coefficients for the three dimensions of 0.89, 0.69–0.77, and 0.25–0.72, respectively. The Cronbach’s alpha values obtained from the sample group in this study is 0.87 for identity dimension, 0.71 for perceptions dimension and 0.68 for reasons dimension. Subscale’s alpha coefficients are as follows; identity A 0.73, identity B 0.79; timeline (acute/chronic) 0.89, timeline (cyclical) 0.59, consequences 0.69, personal control 0.56, treatment control 0.76, illness coherence 0.69, and emotional representations 0.86; personal attributions 0.68, external attributions 0.17, lifestyle attributions.49, uncontrollable bodily attributions 0.014, and chance attributions (single item).

### 2.3. Statistical analysis

The data collected for this study were analyzed using the Statistical Program in Social Sciences (SPSS). Descriptive statistics were used to examine the characteristics of the data, and Pearson correlation analysis was conducted to explore the relationships between variables. For comparisons between the two groups, the independent samples *t*-test was used to test the significance of the difference between the two means. On the other hand, the ANOVA test was used when comparing multiple groups. The Tukey post-hoc test was then applied to identify significant differences between specific groups based on the results of the ANOVA test. The reliability of the scales was assessed using Cronbach’s alpha coefficient. Stepwise regression analysis was employed to identify the predictive factors of the dependent variables.

## 3. Results

As seen in Table 1, a total of 78 cancer patients participated in the study; 53 were female (67.9%) and 25 were male (32.1%). The ages ranged from 19 to 65 years. The total and subscale PTG scores did not differ for sociodemographic variables. The socio-demographic and disease variables are presented in Table 1.

**Table 1:** The socio-demographic and disease variables.

Socio-demographic and disease information	Variable	N	%
Sex	Female	53	67.9
	Male	25	32.1
Age	19–35	7	9.0
	36–50	26	33.3
	51–65	45	57.7
Education status	Literate	2	2.6
	Primary school	26	33.3
	Middle school	8	10.3
	High school	23	29.5
	University and above	19	24.4
Marital status	Married	53	67.9
	Single	14	17.9
	Widow	9	11.5
	Divorced	2	2.6
Employment status	Working	8	10.3
	Not working	63	80.8
	Not working due to illness	7	9.0
How to perceive the economic status	Low	9	11.5
	Middle	52	66.7
	High	18	21.8
Being treatment or control patient status	Treatment patient	15	19.2
	Control patient	63	80.8
Type of cancer	Stomach	4	5.1
	Breast	39	50.0
	Esophagus	1	1.3
	Over	2	2.6
	Lung	11	14.1
	Rectum	6	7.7
	Soft tissue sarcoma	1	1.3
	Pancreas	2	2.6
	Neuroendocrine sarcoma	1	1.3
	Larynx	1	1.3
	Malingnant melanoma	2	2.6
	Thymoma	1	1.3
	Gall bladder	1	1.3
	Osteosarcoma	1	1.3
	Testis	1	1.3
	Anal canal	1	1.3
	Nasopharynx	1	1.3
	Kidney	1	1.3
	Klatskin (Cholangio carcinoma)	1	1.3
Time since diagnosis (months)	6–12	18	23.1
	12–24	29	37.2
	24–48	24	30.8
	48–60	7	9.0
Stage	Stage 1	13	16.7
	Stage 2	33	42.3
	Stage 3	18	23.1
	Stage 4	7	9.0
Presence of similar disease in the family	Yes	31	39.7
	No	47	60.3
Treatments received or currently receiving	Surgery	2	2.6
	Chemotherapy and radiotherapy	9	11.5
	Chemotherapy and surgery	5	6.4
	Radiotherapy and surgery	16	20.5
	Chemotherapy, radiotherapy, and surgery	44	56.4
	Vaccination,chemotherapy, radiotherapy, and surgery	1	1.3
	Vaccination and surgery	1	1.3
Adequacy of knowledge on disease and treatment	Adequate (full)	42	53.8
	Insufficient (partially)	30	38.5
	None at all	6	7.7
How to perceive the seriousness of the disease	Not serious	4	5.1
	A little serious	12	15.4
	Serious	24	30.8
	Quite serious	15	19.2
	Very serious	23	29.5

As seen in Table 2, based on the mean total PTGS scores, the sample group scored above average (M = 69.71, SD = 27.91). The sample group also scored above average on the “Change in Relationships with Others” (*M* = 22.79, SD = 11.00), “Change in Philosophy of Life” (*M* = 13.69, SD = 7.59), and “Change in Self-Perception” (*M* = 33.23, SD = 12.34) subscales.

**Table 2:** The mean scores of total and subscale posttraumatic growth scale.

	*N*	Mean	SD	Min-Max
Posttraumatic growth scale total score (PTGS)	78	69.71	27.91	0–105
Change in relationships with others subscale	78	22.79	11.00	0–35
Change in philosophy of life subscale	78	13.69	7.59	0–25
Change in self-perception subscale	78	33.23	12.34	0–45

Table 3 According to the analysis conducted on the Posttraumatic Growth Scale (PTGS) total score and subscales, significant differences were found based on disease-related variables.

**Table 3:** Comparison of disease related variables with PTGS total and subscale scores.

Posttraumatic growth scale total score					
Adequacy of knowledge on disease and treatment	Source of variation	Sum of squares	SD	Mean square	*F*
	Between groups	5,374,119	2	2,687,059	3,688*
	Within groups	54,645,676	75	728,609	
	Total	60,019,795	77		
	Change in self-perception subscale				
	Source of variation	Sum of squares	SD	Mean square	*F*
	Between groups	911,427	2	455,714	3,159*
	Within groups	10,820,419	75	144,272	
	Total	11,731,846	77		
Time since diagnosis (months)	Change in philosophy of life subscale				
	Source of variation	Sum of squares	SD	Mean square	*F*
	Between groups	518,108	3	172,703	3,261*
	Within groups	3,918,507	74	52,953	
	Total	4,436,615	77		

*p < 0.05.

In terms of the PTGS total score [*F* (2,75) = 3.688] and the “Change in Self-Perception” subscale score [*F* (2,75) = 3.159, *p* < 0.05], there were significant differences among the sample group based on their perception of the adequacy of knowledge on disease and treatment. *Post hoc* (Tukey) tests were conducted to determine which levels differed significantly. The results indicated that individuals who reported having “insufficient (partially)” knowledge about their disease and treatment had significantly higher PTGS total scores compared to those who reported “none” knowledge, at a significance level of 0.05. Specifically, individuals who reported being “insufficient (partially)” knowledgeable about their disease and treatment (Mean = 77.40) had higher PTGS total scores than those who reported having “none” knowledge (Mean = 45.83). Similarly, in terms of the “Change in Self-Perception” subscale, individuals who reported being “insufficient (partially)” knowledgeable (Mean = 36.36) scored higher than those who reported having “none” knowledge (Mean = 23.33).

A significant relationship was found between the “Changes in Philosophy of Life” subscale and the variable of time since diagnosis [*F* (3,74) = 3.261, *p* < 0.05]. *Post hoc* (Tukey) tests were conducted to determine which levels differed significantly. The results indicated that the group with a period of 48–60 months since diagnosis had significantly higher scores on the “Changes in Philosophy of Life” subscale of the PTGS compared to the groups with a period of 6–12 months and 12–24 months, at a significance level of 0.05. Specifically, the group with a period of 48–60 months since diagnosis (Mean = 21.57) had higher scores on the “Changes in Life Philosophy” subscale compared to the groups with a period of 24–48 months (Mean = 12.75) and 6–12 months (Mean = 11.83).

As seen in Table 4, Total PTGS score had moderately positive significant correlation between confrontive coping (*r* = 0.465; *p* < 0.01) self-controlling (*r* = 0.527; *p* < 0.01), seeking social support (*r* = 0.371; *p* < 0.01), accepting responsibility (*r* = 0.533; *p* < 0.01), escape-avoidance (*r* = 0.354; *p* < 0.01), planful problem-solving (*r* = 0.384; *p* < 0.01) subscales, WCI total score (*r* = 0.580; *p* < 0.01), and highly positive correlation with positive reappraisal subscale (*r* = 0.734; *p* < 0.01). Distancing subscale had no significant correlation with PTGS total score.

**Table 4:** Correlation between PTGS total, subscales scores and WCI, IPQ-R total and subscale scores.

Correlations	Posttraumatic growth scale total score	Change in relationships with others subscale	Change in philosophy of life subscale	Change in self-perception subscale
Posttraumatic growth scale total score	1	0.907**	0.846**	0.933**
Change in relationships with others subscale	0.907**	1	0.657**	0.755**
Change in philosophy of life subscale	0.846**	0.657**	1	0.712**
Change in self-perception subscale	0.933**	0.755**	0.712**	1
Confrontive coping	0.465**	0.401**	0.336**	0.487**
Distancing	0.176	0.164	0.146	0.162
Self-controlling	0.527**	0.491**	0.366**	0.529**
Seeking social support	0.371**	0.392**	0.065	0.451**
Accepting responsibility	0.533**	0.513**	0.309**	0.559**
Escape-avoidance	0.354**	0.322**	0.309**	0.320**
Planful problem-solving	0.384**	0.361**	0.299**	0.363**
Positive reappraisal	0.734**	0.593**	0.624**	0.746**
Ways of coping total Score	0.580**	0.529**	0.409**	0.585**
Identity A	−0.003	0.073	−0.011	−0.065
Identity B	−0.070	−0.004	−0.064	−0.116
Timeline (acute-chronic)	−0.262*	−0.252*	−0.217	−0.235*
Timeline (cyclical)	0.153	0.071	0.226*	0.144
Consequences	−0.017	0.020	−0.122	0.018
Personal control	0.098	0.126	0.078	0.062
Treatment control	0.045	0.065	−0.002	0.046
Illness coherence	−0.016	0.059	0.085	−0.140
Emotional represantations	0.075	0.074	0.053	0.072
Perception dimension total score	−0.033	0.019	−0.063	−0.053
Reasons dimension total score	0.108	0.066	0.182	0.074
Personal attributions	0.069	0.009	0.197	0.027
External attributions	0.124	0.132	0.196	0.042
Lifestyle attributions	−0.075	−0.056	−0.067	−0.078
Uncontrollable bodily attribution	0.118	0.020	0.100	0.188
Chance attributions	0.182	0.247*	0.050	0.159

*p < 0.05; **p < 0.01;***p < 0.001.

“Change in Relationships with Others” subscale had moderately positive significant correlation between confrontive coping (*r* = 0.401; *p* < 0.01) self-controlling (*r* = 0.491; *p* < 0.01), seeking social support (*r* = 0.392; *p* < 0.01), accepting responsibility (*r* = 0.513; *p* < 0.01), escape-avoidance (*r* = 0.322; *p* < 0.01), planful problem-solving (*r* = 0.361; *p* < 0.01) positive reappraisal subscale (*r* = 0.593; *p* < 0.01) subscales and WCI total score (*r* = 0.529; *p* < 0.01). Distancing subscale had no significant correlation with “Change in Relationships with Others.”

“Change in Philosophy of Life” subscale had moderately positive significant correlation between confrontive coping (*r* = 0.336; *p* < 0.01) self-controlling (*r* = 0.366; *p* < 0.01), accepting responsibility (*r* = 0.309; *p* < 0.01), escape-avoidance (*r* = 0.309; *p* < 0.01), positive reappraisal subscale (*r* = 0.624; *p* < 0.01) subscales, WCI total score (*r* = 0.409; *p* < 0.01) and marginal moderately positive correlation with planful problem-solving (*r* = 0.299; *p* < 0.01). Distancing and seeking social support subscales had no significant correlation with “Change in Philosophy of Life.”

“Change in Self-Perception” subscale had moderately positive significant correlation between confrontive coping (*r* = 0.487; *p* < 0.01) self-controlling (*r* = 0.529; *p* < 0.01), seeking social support (*r* = 0.451; *p* < 0.01), accepting responsibility (*r* = 0.559; *p* < 0.01), escape-avoidance (*r* = 0.320; *p* < 0.01), planful problem-solving (*r* = 0.363; *p* < 0.01) subscales, WCI total score (*r* = 0.585; *p* < 0.01), and highly positive correlation with positive reappraisal subscale (*r* = 0.746; *p* < 0.01). Distancing subscale had no significant correlation with “Change in Self-Perception.”

According to the Illness Perception Scale-R, Timeline (acute/chronic) subscale had weak and negative significant correlation between PTGS total score (*r* = −262; *p* < 0.05), “Change in Relationships with Others” (*r* = −252; *p* < 0.05), and “Change in Self-Perception” (*r* = −235; *p* < 0.05) subscales. Timeline (cyclical) had weak and positive correlation with “Change in Philosophy of Life” subscale (*r* = 226; *p* < 0.05). From reasons dimension, change attribution subscale had weak and positive correlation between “Change in Relationships with Others” subscale (*r* = 247; *p* < 0.05).

As seen in Table 5, there was a significant relationship between the total and subscale PTGS scores (When the sample group is divided into low and high scores according to the median value) and the total and subscale WCI scores. Accordingly:

**Table 5:** Comparison of posttraumatic growth scale total and subscale scores (Low-High) between ways of coping scale total and subscale scores.

Ways of coping		Change in relationships with others subscale			Change in philosophy of life subscale			Change in self-perception subscale			Posttraumatic growth scale total score		
		*N*	Mean ± SD	*t*	N	Mean ± SD	*t*	*N*	Mean ± SD	*t*	*N*	Mean ± SD	*t*
Confrontive coping	Low	38	7.89 ± 3.96	−2.023*	37	7.89 ± 3.90	−1.976*	37	7.18 ± 3.40	−3.876***	39	7.79 ± 3.78	−2.344*
	High	40	9.52 ± 3.12		41	9.48 ± 3.21		41	10.12 ± 3.27		39	9.66 ± 3.24	
Distancing	Low	38	11.39 ± 4.20	−1.371	37	11.83 ± 4.05	−0.393	37	11.59 ± 4.19	−0.907	39	11.61± 4.21	−0.909
	High	40	12.62 ± 3.71		41	12.19 ± 3.96		41	12.41 ± 3.78		39	12.43± 3.74	
Self-controlling	Low	38	9.50 ± 4.23	−4.103***	37	10.02 ± 4.53	−2.699**	37	9.24 ± 4.32	−4.683***	39	9.69± 4.40	−3.704***
	High	40	13.07 ± 3.43		41	12.51 ± 3.58		41	13.21 ± 3.12		39	12.97± 3.34	
Seeking social support	Low	38	11.28 ± 4.96	−1.651	37	12.13 ± 4.84	−0.034	37	10.64 ± 4.51	−2.903**	39	11.53± 4.99	−1.196
	High	40	12.97 ± 4.01		41	12.17 ± 4.34		41	13.51 ± 4.20		39	12.76± 4.04	
Accepting responsibility	Low	38	6.13 ± 3.07	−3.292**	37	6.51 ± 3.35	−1.969*	37	5.86 ± 2.77	−4.162***	39	6.23± 3.19	−3.032**
	High	40	8.20 ± 2.45		41	7.80 ± 2.40		41	8.39 ± 2.58		39	8.15± 2.34	
Escape-avoidance	Low	37	9.16 ± 3.86	−2.393*	36	9.13 ± 4.53	−2.379*	36	8.91 ± 3.93	−2.886**	39	9.23± 4.40	−2.285*
	High	40	11.32 ± 4.04		41	11.29 ± 3.39		41	11.48 ± 3.87		39	11.30± 3.51	
Planful problem-solving	Low	38	10.21 ± 4.34	−2.077*	37	10.45 ± 4.35	−1.498	37	9.78 ± 4.18	−2.973**	39	10.05± 4.17	−2.502*
	High	40	12.15 ± 3.89		41	11.87 ± 4.00		41	12.48 ± 3.84		39	12.35± 3.96	
Positive reappraisal	Low	38	10.63 ± 5.18	−4.369***	37	10.27 ± 4.96	−5.124***	37	10.16 ± 4.85	−5.415***	39	10.35± 4.80	−5.241***
	High	40	15.02 ± 3.59		41	15.24 ± 3.55		41	15.34 ± 3.55		39	15.4103± 3.6253	
Total	Low	37	103.24 ± 31.77	−3.446**	36	105.52 ± 33.66	−2.643*	36	99.50 ± 30.19	−4.637***	39	103.31± 31.74	−3.527**
	High	40	126.45 ± 27.28		41	123.87 ± 27.20		41	129.17 ± 25.95		39	126.97± 26.98	

*p < 0.05; **p < 0.01;***p < 0.001.

Patients who obtained higher total PTGS scores had higher scores for confrontive coping [*t* (76) = −2.344, *p* < 0.05], self-controlling [*t* (76) = −3.704, *p* < 0.001], accepting responsibility [*t* (76) = −3.032, *p* < 0.01], escape-avoidance [*t* (75) = −2.285, *p* < 0.05], planful problem-solving [*t* (76) = −2.502, *p* < 0.05], positive reappraisal [*t* (76) = −5.241, *p* < 0.001] subscales, and the total WCI score [*t* (75) = −3.52, *p* < 0.01].

Patients who obtained higher scores on the “Change in Relationships with Others” subscale had higher scores in confrontive coping [*t* (76) = −2.023, *p* < 0.05], self-controlling [*t* (76) = −4.103, *p* < 0.001], accepting responsibility [*t* (76) = −3.292, *p* < 0.01], escape-avoidance [*t* (75) = −2.393, *p* < 0.05], planful problem-solving [*t* (76) = −2.077, *p* < 0.05] positive reappraisal [*t* (75) = −4.369, *p* < 0.001] subscales, and the total WCI score [*t* (75) = −3.446, *p* < 0.01].

Patients who obtained higher scores on the “Change in Philosophy of Life” subscale had higher scores in confrontive coping [*t* (76) = −1.976, *p* < 0.05], self-controlling [*t* (76) = −2.699, *p* < 0.01], accepting responsibility [*t* (76) = −1.969, *p* < 0.05], escape-avoidance [*t* (75) = −2.379, *p* < 0.05], positive reappraisal [*t* (76) = −5.124, *p* < 0.001] subscales, and the total WCI score [*t* (75) = −2.643, *p* < 0.05].

Patients who obtained higher scores on the “Change in Self-Perception” subscale had higher scores in confrontive coping [*t* (76) = −3.876, *p* < 0.001], self-controlling [*t* (76) = −4.683, *p* < 0.001], seeking social support [*t* (76) = −2.903 *p* < 0.01], accepting responsibility [*t* (76) = −4.162, *p* < 0.001], escape-avoidance [*t* (75) = −2.886, *p* < 0.01], planful problem-solving [*t* (76) = −2.973, *p* < 0.01], positive reappraisal [*t* (76) = −5.415, *p* < 0.001] subscales, and the total WCI score [*t* (75) = −4.637, *p* < 0.001].

As seen in Table 6, there was a significant relationship between the PTGS subscale scores (When the sample group is divided into low and high scores according to the median value) and the IPQ-R subscale scores. Accordingly:

**Table 6:** Comparison of posttraumatic growth scale subscale scores (Low-High) between illness perception questionnaire subscale scores.

Illness perception Questionnaire-Revised		Change in relationships with others subscale			Change in philosophy of life subscale			Change in self-perception subscale		
		*N*	Mean ± SD	*t*	*N*	Mean ± SD	*t*	*N*	Mean ± SD	*t*
Identity A	Low	38	6.42 ± 3.23	−0.954	37	6.56 ± 3.17	−0.536	37	6.51 ± 3.22	−0.681
	High	40	7.10 ± 3.05		41	6.95 ± 3.13		41	7.00 ± 3.08	
Identity B	Low	38	5.71 ± 3.40	0.211	37	5.56 ± 3.34	−0.151	37	5.62 ± 3.20	−0.016
	High	40	5.55 ± 3.32		41	5.68 ± 3.37		41	5.63 ± 3.49	
Timeline (acute-chronic)	Low	38	18.36 ± 6.24	2.887**	37	17.48 ± 7.10	1.559	37	18.27 ± 6.19	2.660**
	High	40	14.32 ± 6.12		41	15.21 ± 5.72		41	14.51 ± 6.26	
Consequences	Low	38	19.65 ± 5.74	0.700	37	19.29 ± 6.08	0.139	37	18.32 ± 5.64	−1.339
	High	40	18.77 ± 5.38		41	19.12 ± 5.09		41	20.00 ± 5.40	
Personal control	Low	38	21.68 ± 3.92	−1.596	37	22.27 ± 3.89	−0.273	37	21.78 ± 4.28	−1.332
	High	40	23.07 ± 3.77		41	22.51 ± 3.92		41	22.95 ± 3.44	
Treatment control	Low	38	22.10 ± 2.53	−0.451	37	22.70 ± 2.31	1.310	37	22.16 ± 2.72	−0.274
	High	40	22.40 ± 3.18		41	21.85 ± 3.26		41	22.34 ± 3.02	
Illness coherence	Low	38	18.55 ± 3.90	−1.036	37	18.81 ± 4.36	−0.497	37	18.91 ± 4.19	−0.285
	High	40	19.55 ± 4.55		41	19.29 ± 4.19		41	19.19 ± 4.35	
Timeline (cyclical)	Low	38	11.05 ± 3.80	−1.034	37	11.45 ± 3.80	−0.140	37	10.27 ± 3.85	−2.788**
	High	40	11.97 ± 4.06		41	11.58 ± 4.10		41	12.65 ± 3.70	
Emotional represantations	Low	38	17.73 ± 6.89	−0.106	37	17.16 ± 7.58	−0.815	37	16.62 ± 7.32	−1.499
	High	40	17.90 ± 6.71		41	18.41 ± 5.96		41	18.90 ± 6.10	
Perception dimension total score	Low	38	129.15 ± 15.24	0.352	37	129.18 ± 16.26	0.361	37	126.35 ± 15.52	−1.291
	High	40	128.00 ± 13.80		41	128.00 ± 12.76		41	130.56 ± 13.26	
Personal attributions	Low	38	19.28 ± 5.45	−0.246	37	18.48 ± 5.43	−1.375	37	18.94 ± 5.63	−0.721
	High	40	19.62 ± 6.51		41	20.34 ± 6.37		41	19.92 ± 6.31	
External attributions	Low	38	8.57 ± 2.75	−1.007	37	8.24 ± 2.73	−2.057*	37	8.59 ± 2.32	−0.932
	High	40	9.20 ± 2.69		41	9.48 ± 2.60		41	9.17 ± 3.04	
Lifestyle attributions	Low	38	6.23 ± 2.67	0.091	37	6.67 ± 3.06	1.339	37	6.13 ± 3.08	−0.197
	High	40	6.17 ± 3.24		41	5.78 ± 2.84		41	6.26 ± 2.88	
Uncontrollable bodily attribution	Low	38	6.89 ± 2.49	0.348	37	6.48 ± 2.61	−1.054	37	6.24 ± 2.12	−1.916*
	High	40	6.70 ± 2.45		41	7.07 ± 2.30		41	7.29 ± 2.64	
Chance attributions	Low	38	2.05 ± 1.46	−2.920**	37	2.32 ± 1.51	−1.201	37	2.48 ± 1.59	−0.289
	High	40	3.00 ± 1.39		41	2.73 ± 1.48		41	2.58 ± 1.43	
Reasons dimension total score	Low	38	43.05 ± 9.54	−0.721	37	42.21 ± 9.83	−1.412	37	42.40 ± 9.16	−1.249
	High	40	44.70 ± 10.57		41	45.41 ± 10.12		41	45.24 ± 10.72	

*p < 0.05; **p < 0.01;***p < 0.001.

Patients who obtained lower scores on the “Change in Relationships with Others” subscale had higher timeline (acute/chronic) scores [*t* (76) = 2.887, *p* < 0.01], and those who obtained higher scores on the “Change in Relationships with Others” subscale had higher change attribution scores [*t* (76) = −2.920, *p* < 0.01].

Patients who obtained higher scores on the “Change in Philosophy of Life” subscale had higher in external attribution scores [*t* (76) = −2.057, *p* < 0.05].

Patients who obtained lower scores on the “Change in Self-Perception” subscale had higher timeline (acute/chronic) [*t* (76) = 2.660, *p* < 0.01], timeline (cyclical) [*t* (76) = −2.788, *p* < 0.01], and uncontrollable bodily attributions [*t* (76) = −1.916, *p* < 0.05] scores.

In the stepwise regression analysis reported in Table 7, the order in which the subscales were included in the analysis was: confrontive coping, distancing, self-controlling, seeking social support, accepting responsibility, escape-avoidance, planful problem-solving, positive reappraisal, identity A, identity B, timeline (acute/chronic), timeline (cyclical), consequences, personal control, treatment control, illness coherence and emotional representations, personal attributions, external attributions, lifestyle attributions, uncontrollable bodily attributions, and chance attributions.

**Table 7:** Predictors of posttraumatic growth scale total and subscale scores.

Posttraumatic growth scale total score										
							Change statistics			
**Step**	**Variable**	***B***	**Beta**	***R* Square**	**Adjusted *R* Square**	***F***	***R* Square Change**	***F* Change**	**SD**	**Sig *F* Change**
1	Positive reappraisal	4.080	0.715	0.511	0.504	78.290***	0.511	78.290	1,75	0.001
2	Distancing	−1.661	−0.236	0.556	0.544	46.311***	0.045	7.523	2,74	0.008
3	Self-controlling	2.188	0.339	0.621	0.605	39.814***	0.065	12.467	3,73	0.001

**Change in relationships with others subscale**										
							**Change statistics**			
**Step**	**Variable**	***B***	**Beta**	***R* Square**	**Adjusted *R* Square**	***F***	***R* Square Change**	***F* Change**	**SD**	**Sig *F* Change**
1	Positive reappraisal	1.323	0.578	0.334	0.325	37.607***	0.334	37.607	1,75	0.001
2	Accepting responsibility	0.904	0.238	0.371	0.354	21.855***	0.037	4.399	2,74	0.039
3	Distancing	−0.705	−0.250	0.417	0.393	17.369***	0.045	5.652	3,73	0.020
4	Self-controlling	0.771	0.298	0.463	0.433	15.531***	0.047	6.260	4,72	0.015

**Change in philosophy of life subscale**										
							**Change statistics**			
**Step**	**Variable**	***B***	**Beta**	***R* Square**	**Adjusted *R* Square**	***F***	***R* Square Change**	***F* Change**	**SD**	**Sig *F* Change**
1	Positive reappraisal	0.945	0.603	0.364	0.355	42.897***	0.364	42.897	1,75	0.001
2	Seeking social support	−0.422	−0.258	0.418	0.402	26.557***	0.054	6.863	2,74	0.011
3	Escape-avoidance	0.417	0.228	0.462	0.440	20.881***	0.044	5.966	3,73	0.017
4	Illness coherence	0.354	0.202	0.501	0.473	18.058***	0.039	5.621	4,72	0.020

**Change in self-perception subscale**										
							**Change statistics**			
**Step**	**Variable**	***B***	**Beta**	***R* Square**	**Adjusted *R* Square**	***F***	***R* Square Change**	***F* Change**	**SD**	**Sig *F* Change**
1	Positive reappraisal	1.812	0.725	0.526	0.520	83.174***	0.526	83.174	1,75	0.001
2	Distancing	−0.835	−0.271	0.585	0.574	52.244***	0.060	10.632	2,74	0.002
3	Self-controlling	0.999	0.354	0.656	0.642	46.367***	0.070	14.936	3,73	0.001
4	Accepting responsibility	0.877	0.212	0.680	0.663	38.329***	0.025	5.549	4,72	0.021

*p < 0.05; **p < 0.01;***p < 0.001.

As seen in Table 7, when three WCI subscales, namely positive reappraisal, distancing, and self-controlling, were included in the analysis, the total variance explained by these variables was 62%, and this was significant [*F* (3,73) = 39.814, *p* < 0.001]. Other variables that were not predictive of the total scores of the dependent variable were excluded fro*M* the regression analysis. When the contribution of these three variables in explaining the variance of the dependent variable was analyzed, positive reappraisal explained 51% of the variance [*F* (1,75) = 78.290, *p* < 0.001]; when distancing was included in the analysis, the variance explained increased to 55% [*F* (2,74) = 46.311, *p* < 0.001] and distancing negatively predicted PTG. When self-controlling was included in the analysis, the variance increased to 62% [*F* (3,73) = 39.814, *p* < 0.001].

When four of the WCI subscales, namely positive reappraisal, accepting responsibility, distancing, and self-controlling, were included in the analysis, the total variance explained by these variables was 46% and this was significant [*F* (4,72) = 15.531, *p* < 0.001]. Other variables that were not predictive of the “Change in Relationships with Others” subscale were excluded fro*M* the regression analysis. Among the four coping ways, positive reappraisal was found to explain the highest amount of variance, accounting for 33% of the total variance [*F* (1,75) = 37.607, *p* < 0.001]. When accepting responsibility was added into the analysis, the amount of variance explained increased to 37% [*F* (2,74) = 21.85, *p* < 0.001], while distancing was found to negatively affect “Change in Relationships with Others” and increased the amount of variance explained to 41% [*F* (3,73) = 17.369, *p* < 0.001]. Finally, when self-controlling was included in the analysis, the total amount of variance explained reached 46% [*F* (4,72) = 15.531, *p* < 0.001].

When three subscales of the WCI, namely positive reappraisal, seeking social support, and escape-avoidance, and the illness coherence subscale of the IPQ-R, were included in the analysis, the total variance explained by these variables was 50%, and this was significant [*F* (4,72) = 18.058, *p* < 0.001]. Other variables that were not predictive of the “Change in Philosophy of Life” subscale were excluded fro*M* the regression analysis. When the contribution of these four variables in explaining the variance of the “Change in Philosophy of Life” subscale was examined, it was seen that positive reappraisal explained 36% of the variance [*F* (1,75) = 42.897, *p* < 0.001], and when seeking social support was included in the analysis, the variance explained increased to 41% [*F* (2,74) = 26.557, *p* < 0.001], and seeking social support predicted “Change in Philosophy of Life” negatively. When escape-avoidance was included in the analysis, the variance explained increased to 46% [*F* (3,73) = 20.881, *p* < 0.001], and when illness consistency was included, the variance explained increased to 50% [*F* (4,72) = 18.058, *p* < 0.001].

When four subscales of the WCI, namely positive reappraisal, distancing, self-controlling, and accepting responsibility, were included in the analysis, the total variance explained by these variables was 68% and this was significant [*F* (4,72) = 38.329, *p* < 0.001]. Other variables that were not predictive of the “Change in Self-Perception” subscale were excluded fro*M* the regression analysis. When the contribution of these four variables in explaining the variance in “Change in Self-Perception” was examined, positive reappraisal explained 52% of the variance [F (1,75) = 83.174, *p* < 0.001], and when distancing was included in the analysis, the variance explained increased to 58% [F (2,74) = 52.244, *p* < 0.001] and distancing predicted the change in self-perception negatively. When self-controlling was included in the analysis, the variance explained increased to 65% [F (3,73) = 46.367, *p* < 0.001], and when accepting responsibility was included, the variance increased to 68% [F (4,72) = 38.29, *p* < 0.01].

The essential first-order causes of the participants’ diseases can be seen in Table 8 and were found to be: 11 patients (14.1%) smoking-alcohol-nutritional problems, 12 patients (15.4%) environmental factors (Pollution, Chernobly, Virus,…), 4 patients (5.1%), hereditary, 36 patients (46.2%) stress-overwork, 5 patients (6.4%) emotional problems, 3 patients (3.8%) fate, 2 patients (2.6%) family problems, 1 patient (1.3%) aging, 2 patients (2.6%) sadness due to the Marmara earthquake, 1 patient (1.3%) playing with the skin (self-doctoring), 1 patient (1.3%) not being able to breastfeed.

**Table 8:** The results regarding the reasons of cancer.

Reasons (1)	N	%	Reasons (2)	N	%	Reasons (3)	N	%
Smoking- alcohol- nutritional problems	11	14.1	Smoking alcohol- nutritional problems	15	19.2	Smoking alcohol- nutritional problems	7	9.0
Enviromental factors (Pollution, Chernobly, Virus,…)	12	15.4	Enviromental Factors (Pollution, Chernobly, Virus,…)	9	11.6	Enviromental Factors (Pollution, Chernobly, Virus, Food from the Black Sea region)	17	21.8
Hereditary	4	5.1	Hereditary	8	10.3	Hereditary	7	9.0
Stress-overwork	36	46.2	Stress-overwork	14	18	Stress-overwork	13	16.7
Emotional problems	5	6.4	Emotional problems	6	7.7	Emotional problems	6	7.7
Fate	3	3.8	Fate	7	9.0	Fate	1	1.3
Family problems	2	2.6	Family problems	4	5.1	Family problems	6	7.7
Attributed to aging	1	1.3	Attributed to aging	1	1.3	Attributed to aging	2	2.6
Sadness due to the Marmara eartquake	2	2.6	Sadness due to the Marmara eartquake	1	1.3	Early menarche	1	1.3
Playing with the skin (self-doctoring)	1	1.3	Life style/Philosophy (e.g. Not being able to live in the moment)	4	5.1	Life style/philosophy, personal traits	9	11.5
Not being able to breastfeed	1	1.3	Medical negligence	4	5.1	Medical negligence	2	2.6
–	–	–	Decreased body resistance	5	6.4	Decreased body resistance	7	9.0

The second most important causes of disease were found to be: 15 patients (19.2%) smoking- alcohol-nutritional problems, 9 patients (11.6%) environmental factors (Pollution, Chernobly, Virus,…), 8 patients (10.3%), hereditary, 14 patients (18%) stress-overwork, 6 patients (7.7%) emotional problems, 7 patients (9.0%) fate, 4 patients (5.1%) family problems, 1 patient (1.3%) aging, 1 patient (1.3%) sadness due to the Marmara earthquake, 4 patients (5.1%) life style/philosophy (e.g., Not being able to live in the moment), 4 patients (5.1%) medical negligence, 5 patients (6.4%) decreased body resistance.

The third most important causes of disease were seen to be: 7 patients (9.0%) smoking-alcohol- nutritional problems, 17 patients (21.8%) environmental factors (Pollution, Chernobly, Virus, Food from the Black Sea region), 7 patients (9.0%), hereditary, 13 patients (16.7%) stress-overwork, 6 patients (7.7%) emotional problems, 1 patient (1.3%) fate, 6 patients (7.7%) family problems, 2 patient (2.6%) aging, 1 patient (1.3%) early menarche, 9 patients (11.6%) life style/philosophy/personal traits, 2 patients (2.6%) medical negligence, 7 patients (9.0%) decreased body resistance.

## 4. Discussion

In this study, which examined PTG in cancer patients within the scope of ways of coping and illness perceptions, no significant differences were found for PTG scores in terms of the basic socio-demographic variables (age, gender). This supports related literature that found no significant difference in terms of variables such as gender and age in relation to satisfaction with life, PTG, coping strategies, and resilience in cancer survivors (Adamkovic et al., 2022), and no significant differences between the PTG of female and male patients in the Predicting PTG Based on Coping Strategies in Women and Men Involved with Advanced Cancer study (Baghjari et al., 2017). However, our findings contrast research (Dobrikova et al., 2021) showing that female patients undergoing cancer treatment and in the last stage of the disease have significantly higher PTG (new possibilities, spiritual change subscales) than male patients in the same circumstances. Studies with cancer patients, who are a vulnerable and sensitive group, may yield statistically insignificant results when sample sizes are insufficient for subgroups based on variables such as age, gender, and cancer type. This situation often arises due to the challenging data collection process with sensitive this vulnerable group.

It was observed that the sample had above-average total scores for the PTGS (M: 69.72, Min.-Max.: 0–105) as well as for the three PTGS subscales: “Change in Relationships with Others,” (M: 22.79, Min.-Max.: 0–35) “Change in Life Philosophy,” (M: 13.69, Min.-Max.: 0–25) and “Change in Self-Perception.” (M: 33.23, Min.-Max.: 0–45) The total and subscale PTGS scores were higher like in similar studies. For example, average PTG scores have been reported as 64.1 for breast cancer patients (Cordova et al., 2001), 54.2 for mixed-type cancer patients (Lechner et al., 2003), 46.6 for prostate cancer patients (Thornton and Perez, 2006), 47.8 for recurrent breast cancer patients (Ho et al., 2004), 64.67 for cancer patients after bone marrow transplantation (Widows et al., 2005), 68.09 for survivors of ovarian cancer (Oh et al., 2021), 65.68 for women diagnosed with breast cancer (Aydoğdu and Dirik, 2021), and 48.33 (Liu et al., 2020), and 69.01 (Li, 2022) for breast cancer patients.

Most previous studies used the five subscales of the PTGS. However, this study utilized the three-subscale version, which may be why the sample group experienced a higher level of PTG like in other studies. This finding is consistent with the existing literature. It is important to note that the PTG scores in this study are highly dependent on the study population and methodology, and should not be used to generalize across different groups. Additionally, individual scores may vary widely depending on various factors, such as stage and type of cancer, treatment experiences, and personal characteristics.

In the analysis examining whether disease-related variables differed based on the total and subscale scores of PTGS, significant results were obtained only for the variables “Time since diagnosis (months)” and “Adequacy of knowledge on disease and treatment.” The level of posttraumatic growth in cancer patients has been found to have a significant relationship only with the “Changes in Philosophy of Life” subscale of the Posttraumatic Growth Scale. According to this relationship, patients who have passed 48–60 months since diagnosis have higher scores on the Changes in Philosophy Life subscale than those who have passed 6–12 and 24–48 months. In a study conducted by Sears et al. (2003) on early-stage breast cancer patients, the time elapsed since diagnosis was found to be associated with posttraumatic growth. It was revealed that a more extended time elapsed from diagnosis was a predictive factor for higher overall scores in posttraumatic growth. In a study on posttraumatic growth and the time elapsed since diagnosis, it is suggested that it may be stronger following diagnosis or the completion of treatment compared with after 1 or 2 years. This is attributed to individuals becoming accustomed to the disease over time and becoming automatic (Stanton et al., 2006). In their longitudinal study on breast cancer patients, Manne et al. (2004) discuss a consistent and significant increase in posttraumatic growth scores after approximately 18 months. Cordova et al. (2001) found that the time elapsed since breast cancer diagnosis was positively associated with posttraumatic growth in participants who had been diagnosed with cancer for 5 years or less and had completed their treatments at least 2 months ago. Weiss (2004) examined posttraumatic growth in spouses of breast cancer patients and included individuals diagnosed 1–5.5 years ago, with a time elapsed since diagnosis ranging from 15 to 66 months. The time elapsed since diagnosis was weakly correlated with spouses’ posttraumatic growth scores, and a shorter time since diagnosis was positively correlated with posttraumatic growth scores. Lechner et al. (2003) did not find a significant difference in the benefit of time elapsed since diagnosis in cancer patients. Widows et al. (2005) also found no significant difference in the time elapsed since bone marrow transplantation. The studies mentioned above do not provide a direct comparison opportunity for the “Changes in Philosophy of Life” subscale of the posttraumatic growth scale because they were either included in the five subscales of their analyses or based on total scores. However, as seen, there are studies conducted with patients who have passed 5 years (Ho et al., 2004), generally considering approximately 12–18 months as a high probability for posttraumatic growth to occur. In this study, however, it is observed that the group with a time elapsed of 48–60 months receives higher scores in the Changes in Philosophy of Life subscale. This may be attributed to philosophical change requiring a long process of adaptation and integration.

Analyzed based on the variable of the adequacy of knowledge about the disease and treatment, a significant relationship was found between the “Total Score of Posttraumatic Growth Scale” and the “Change in Self-perception” subscale. It was observed that those who indicated “insufficient (partially)” knowledge about their disease and treatment received higher scores compared to those who indicated “none” in terms of the “Total Score of Posttraumatic Growth.” Similarly, a significant relationship was found between those who indicated “insufficient (partially)” and “none” in the “Change in Self-perception” subscale, where those who indicated “insufficient (partially)” received higher scores compared to those who indicated “none.” These results indicate that even partially knowing their illness enables patients to achieve higher posttraumatic growth scores than not knowing at all. As stated by Armay (2006), individuals’ levels of knowledge about their diseases can be determining factors in their reactions. Adequacy of knowledge can facilitate coping, eliminate catastrophic perceptions, and positively affect reactions related to the illness.

On the other hand, knowledge inadequacy can lead to increased anxiety, difficulties in treatment adherence, and delayed recovery. In light of this information, the finding is consistent with the knowledge that being partially knowledgeable about the disease can eliminate catastrophic perceptions and lead to positive reactions, compared to not knowing. Also, according to Boyacıoğlu et al. (2022), higher knowledge about disease positively affects PTG.

Regarding the correlation analysis, total PTGS and subscale scores had a moderately positive significant correlation between WCI total and subscale scores except “distancing.” Moreover, the “seeking social support” subscale had no significant correlation with “Change in Philosophy of Life. According to the Posttraumatic Growth (PTG) model proposed by Tedeschi and Calhoun, coping stands out as a fundamental element. It has been argued that coping and posttraumatic growth are synonymous in the history of the concept (Calhoun and Tedeschi, 1998). This study also reveals a high positive correlation between positive coping ways and PTG. Baghjari et al. (2017) found no correlation between emotion-focused coping and PTG, while Baník et al. (2022) stated that existential factors (such as religious beliefs) associated with PTG have a linear, while posttraumatic stress symptoms have a curvilinear correlation. Schmidt et al. (2012) demonstrated in their study involving 54 cancer patients that active coping, positive reframing, and religion are associated with PTG. Similarly, Jaafar et al. (2021) established that approach coping strategies are related to PTG.

According to the Illness Perception Questionnaire-R, Timeline (acute/chronic) subscale had a weak and negative significant correlation between PTGS total score “Change in Relationships with Others,” and “Change in Self-Perception” subscales. Timeline (cyclical) had a weak and positive correlation with the “Change in Philosophy of Life” subscale. Fro*M* the reasons dimension, the change attribution subscale had a weak and positive correlation with the “Change in Relationships with Others” subscale. It is known that individuals’ sense of having control over traumatic events and attributions to the occurrence of traumatic events are important in terms of growth and psychopathology (Şalcıoğlu, 2003; Cao et al., 2018). In our study, there is a weak and negative correlation between variables, and it is believed that the sense of being able to achieve well-being after the illness is associated with posttraumatic growth. There is limited literature on illness perception and posttraumatic growth. However, Rahimzadegan et al. (2022) reported that negative illness perception is negatively correlated with posttraumatic growth, while positive illness perception is positively correlated in cancer patients. Rogan et al. (2013) stated that a firm belief in the controllability of symptoms associated with brain damage and adaptive coping strategies are related to posttraumatic growth.

The study also examined whether there was a significant difference between ways of coping and illness perceptions based on grouping the total and subscale PTG scores into “Low” and “High” categories. It was observed that all except the distancing subscale of the WCI, differed relative to the total or at least one of the PTGS subscales in favor of those with high scores (Please refer to Table 3 for the mean values in favor). Rogan et al. (2013) found that adaptive coping strategies were associated with higher levels of PTG in individuals diagnosed with acquired brain injury. Baghjari et al. (2017) reported that cognitive appraisal and seeking social support among problem-focused coping strategies explained PTG in advanced cancer patients, suggesting that clinical interventions such as problem-focused coping skills training and facilitating social support could be beneficial. Lelorain et al. (2012) conducted a qualitative study and revealed that PTG was a specific theme for women who possessed high levels of coping skills. Previous studies generally demonstrate that the use of functional coping strategies increases PTG, and even escape-avoidance coping strategies can become functional and effective in PTG. Fro*M* this perspective, our findings regarding coping strategies are consistent with the literature and indicate that coping strategies are an important variable in PTG.

Regarding the IPQ-R (illness perceptions), no significant difference was found relative to the total PTGS scores. For the PTGS subscales, those who scored higher on the “Change in Philosophy of Life” subscale had higher external attribution scores than those who scored lower. When the “Change in Self-Perception” subscale was analyzed, timeline (acute-chronic) scores differed in favor of those with low scores; likewise, the timeline (cyclical) and uncontrollable bodily attribution subscales also differed in favor of those with high scores. There was differentiation based on the timeline (acute-chronic) factor in favor of those who scored low on the “Change in Relationships with Others” subscale. In terms of the causes of illness, attributing the illness to chance factors created a difference in “Change in Relationships with Others.” It was observed that individuals who scored high on this subscale tended to attribute their illness more to chance factors compared to those who score low (Please refer to Table 4 for the mean values in favor).

In recent years, only a few studies have considered both PTG and illness perceptions together. For example, in Rogan et al.’s (2013) study of individuals who had acquired brain injury, there was no significant correlation between illness perceptions and PTG; the only result was that higher levels of PTG were associated with increased utilization of stronger beliefs about treatment-induced controllability (*r* = 0.263). Furthermore, Lau et al. (2018) examined the relationship between illness perceptions and PTG in newly diagnosed HIV-positive men. Linear regression analyses indicated that the emotional representation subscale and five cognitive representation subscales (timeline, consequences, identity, God’s punishment/will, and luck/chance attribution) were negatively associated with PTG. However, another four cognitive representations (coherence, treatment control, personal control, and attribution to carelessness) were positively associated with PTG. The emotional representation entirely mediated the relationships between the five cognitive representation subscales and PTG. Rahimzadegan et al.’s (2022) study of PTG and illness perceptions in cancer patients, showed that negative illness perceptions had a significant and negative relationship with PTG, while optimistic illness perceptions had a significant and positive relationship with PTG (*p* < 0.05). Taken together, these results indicate the need for interventions that promote PTG in cancer patients, particularly interventions that target illness perceptions, especially emotional representation.

According to the results of the stepwise regression analysis conducted to determine what variables predicted the total PTG score, three WCI subscales––positive reappraisal, distancing, and self-controlling––were found to have predictive effects. It was observed that distancing negatively predicted PTG, while “Change in Relationships with Others” was predicted by a combination of positive reappraisal, accepting responsibility, distancing, and self-controlling, but negatively predicted by distancing. The combined variables of positive reappraisal, seeking social support, escape-avoidance, and illness coherence predicted “Change in Philosophy of Life,” while seeking social support negatively predicted “Changes in Philosophy of Life.” “Change in Self-Perception” was predicted by positive reappraisal, distancing, self-controlling, and accepting responsibility, but negatively predicted by distancing.

Oh et al. (2021) found that religion, ways of coping and posttraumatic stress symptoms explained 52.2% of PTG in ovarian cancer survivors. Baghjari et al. (2017) found that problem-oriented coping strategies, cognitive assessment, and seeking social support explained 53% of PTG changes (*p* < 0.001) in advanced cancer patients; however, emotion-oriented strategies, including emotional inhibition and somatic inhibition, had no significant relation with PTG, while the regression model revealed that using problem-oriented strategies can predict the rate of PTG. Schmidt et al. (2012), in a cancer patient regression analysis, suggested that using positive reframing and religion as coping strategies may mediate the relationship between secure attachment and PTG. Widows et al. (2005), in a study exploring PTG after bone marrow transplantation, found that greater use of positive reappraisal, problem-solving, and seeking alternative rewards coping methods before transplantation was associated with higher PTG. Ho et al. (2004) found that positive coping was the most crucial predictor of PTG compared to negative coping in a study of cancer survivors in China. Sears et al. (2003), in their study of patients with early-stage breast cancer, found that positive coping benefited PTG and coping styles are interconnected but separate processes; they also found that seeking more social support was associated with PTG. Thornton and Perez (2006) investigated PTG in prostate cancer survivors and their spouses, in terms of coping 1 year after surgery; having a positive approach and using emotional support were found to be associated with PTG for both the patients and their spouses. In different studies dealing with different variables, the predictors of PTG emerge as variables with similar dynamics. As seen in the studies above, variables such as seeking social support, religious coping, confronting the problem are important in the predictors of PTG.

The results of both the independent group t-test analysis and the regression analysis of the ways of coping and illness perceptions data showed especially that ways of coping are essential for cancer patients to experience positive change. This study and previous studies reveal that coping mechanisms such as confrontation, positive reappraisal, and seeking social support are particularly important. In terms of illness perceptions, there have been a limited number of studies in the field, but when this and other studies (e.g., Rogan et al., 2013; Lau et al., 2018; Rahimzadegan et al., 2022) are examined, it can be seen that attributions regarding the timeline of the disease, reasons, and idea on the disease prognosis are important in PTG. Considering that these variables are related to the cognitive evaluation processes of individuals and cognitive ways of coping, especially positive reappraisal and problem-solving, this is an important finding. It is thought that strengthening positive ways of coping in approaching cancer patients may also positively affect the disease perception process. Finally, the fact that similar findings were obtained in this study of data collected in 2007 and previous studies published before 2022 strengthens the consistency of this information and the necessity of focusing on ways of coping in interventions.

When patients in the sample group were asked about the three most important causes of their cancer, the first reason given was stress/overwork at 46.2%, the second was Smoking Alcohol- Nutritional problems at 19.2%, and the third was environmental factors (Pollution, Chernobly, Virus, Food from the Black Sea region at 21.8%). Smoking, overwork, nutritional problems, fate, bad luck, personality traits, etc. were also seen to be causal issues. Some of the unique answers given were: Chernobyl-radiation (in terms of patients living in the Black Sea region), earthquake-related sadness, playing with the skin (self-doctoring), not being able to breastfeed, keeping problems inside, medical negligence, not being able to live in the moment/self, foods from the Black Sea region, and early menarche.

References to geographical and environmental events such as earthquakes and the Chernobyl disaster were relatively high. In addition, culture-specific attributions, such as not being able to breastfeed children and having early menarche, stood out among women. The not being able to live in the moment/not being able to live oneself reason for getting cancer was an interesting finding. As Yalom (2002) states, “Most cancer patients know that they are living more fully now, they are no longer postponing their lives to a future period. The individual realizes that they can only live in the present moment.” (pp. 263). This finding can be interpreted as a punishment/reward for not being able to “exist” before having cancer.

## 5. Limitations

Although the present study contributes to the existing literature on PTG in cancer patients, particularly for the Turkish population, it has several limitations. Firstly, the study employed a cross-sectional design, which limited the ability to establish causal relationships between variables. A longitudinal design would be more appropriate for exploring the precise relationships between variables. Secondly, one of the main limitations of the study was the small sample size. However, considering the challenging nature of collecting data from special groups like cancer patients (e.g., obtaining participant consent, scheduling interviews during suitable physical health conditions, etc.), even data from a single individual can be considered valuable. However, this study compared 2007 data with the findings of current publications on the topic and observed similarities in results across different sample sizes. This provides support for the study despite its limited scope. Eventually, the limitation is that some subscales in the reasons dimension of the Illness Perception Questionnaire has low reliability coefficients. However, the scale has been adapted in Turkey and is used as a reliable measure. The low reliability coefficients of the subscales may be due to the small number of items, typically 2–3, in those subscales.

## 6. Conclusion

Receiving a cancer diagnosis and living with this disease is an inevitable and traumatic reality. This traumatic event affects the patient and many individuals who witness the process, including their loved ones. This impact can be damaging, such as the development of psychiatric disorders. However, it can also manifest positively under the concept of “posttraumatic growth,” which has gained increasing importance in the literature and continues to do so. Examining ways of coping and individuals’ perceptions of their traumatic experiences, which are believed to be influential in positive growth following traumatic experiences, is particularly important for developing intervention approaches. The critical point in these intervention approaches is to focus on the perception of the illness and ways of coping to keep hope alive in patients. In addition, since each type of cancer has its dynamics (for example, breast cancer is related to the image of femininity) and process (stage and course of the disease), these features should be considered in individual intervention approaches and group work.

This study investigated PTG, ways of coping, and illness perceptions in cancer patients, highlighting the need to strengthen positive coping methods and implement interventions that target the cognitive aspects of their illness perceptions. This study was one of the first to explore PTG in cancer patients in Turkey. Considering the culture-specific differences in illness experiences, the emergence of similar findings to studies of the same topic in different cultures and at different times underscores the significance of the findings. This sheds light on the importance of ways of coping and PTG for our understanding of other traumatic experiences, particularly those related to other chronic illnesses.

## Data availability statement

The raw data supporting the conclusions of this article will be made available by the authors, without undue reservation.

## Ethics statement

The studies involving humans were approved by the Istanbul University Medical Faculty. The studies were conducted in accordance with the local legislation and institutional requirements. The participants provided their written informed consent to participate in this study.

## Author contributions

All authors listed have made a substantial, direct, and intellectual contribution to the work and approved it for publication.

## Conflict of interest

The authors declare that the research was conducted in the absence of any commercial or financial relationships that could be construed as a potential conflict of interest.

## Publisher’s note

All claims expressed in this article are solely those of the authors and do not necessarily represent those of their affiliated organizations, or those of the publisher, the editors and the reviewers. Any product that may be evaluated in this article, or claim that may be made by its manufacturer, is not guaranteed or endorsed by the publisher.
